# The advantages of mapping slow brain potentials using DC‐coupled graphene micro‐transistors: Clinical and translational applications

**DOI:** 10.1002/ctm2.968

**Published:** 2022-07-08

**Authors:** Rob. C Wykes, Eduard Masvidal‐Codina, Anton Guimerà‐Brunet, Jose. A Garrido

**Affiliations:** ^1^ Department of Clinical & Experimental Epilepsy UCL Queen Square Institute of Neurology London United Kingdom; ^2^ Nanomedicine Lab University of Manchester Manchester United Kingdom; ^3^ Catalan Institute of Nanoscience andNanotechnology (ICN2) CSIC and The Barcelona Institute of Science and Technology (BIST), Campus UAB Bellaterra Barcelona Spain; ^4^ Centro de Investigación Biomédica en Red de Bioingeniería Biomateriales y Nanomedicina (CIBER‐BBN) Instituto de Salud Carlos III Madrid Spain; ^5^ Institut de Microelectrònica de Barcelona IMB‐CNM (CSIC) Esfera UAB Bellaterra Spain; ^6^ ICREA Barcelona Spain

There is growing interest in examining oscillations and brain signals outside traditional EEG bands (0.3–80 Hz), as these regimes contain useful electrographic biomarkers for the diagnosis, monitoring and prognosis of neurological disorders and injuries.^1^ These include high gamma (80–200 Hz), ripples and high‐frequency oscillations (HFOs) (200–500 Hz), as well as infraslow oscillations (<0.1 Hz) and ultraslow potential shifts (UPS). In particular, UPS have remained poorly explored in clinical settings with the notable exception of the Co‐Operative Studies on Brain Injury Depolarizations (COSBID) consortium,^2^ and specialist epilepsy surgical centres.^3^ This is due to the associated technical difficulties recording such slow potentials that require DC‐coupled amplifiers and highly stable electrodes. However, UPS include clinically relevant events including preseizure DC shifts (1–3 mV), and large (tens of millivolt) spreading depolarisations (SD) which are thought to play an important role in brain injury and contribute to the pathophysiology associated with migraine with aura, stroke and epilepsy.^4^ Therefore, the ability to record and map a wide range of brain signals, from UPS to single units, using the same electrophysiological array will greatly advance our understanding of brain diseases and aid the clinical management of patients with diverse neurological disorders and injuries. Therefore, development of improved electrophysiological devices capable of detecting and mapping wide bandwidth signals with high‐fidelity and spatial resolution is warranted.^5^, ^6^

## 1 WHAT ARE GRAPHENE MICRO‐TRANSISTORS – HOW DO THEY WORK?

**FIGURE 1:**
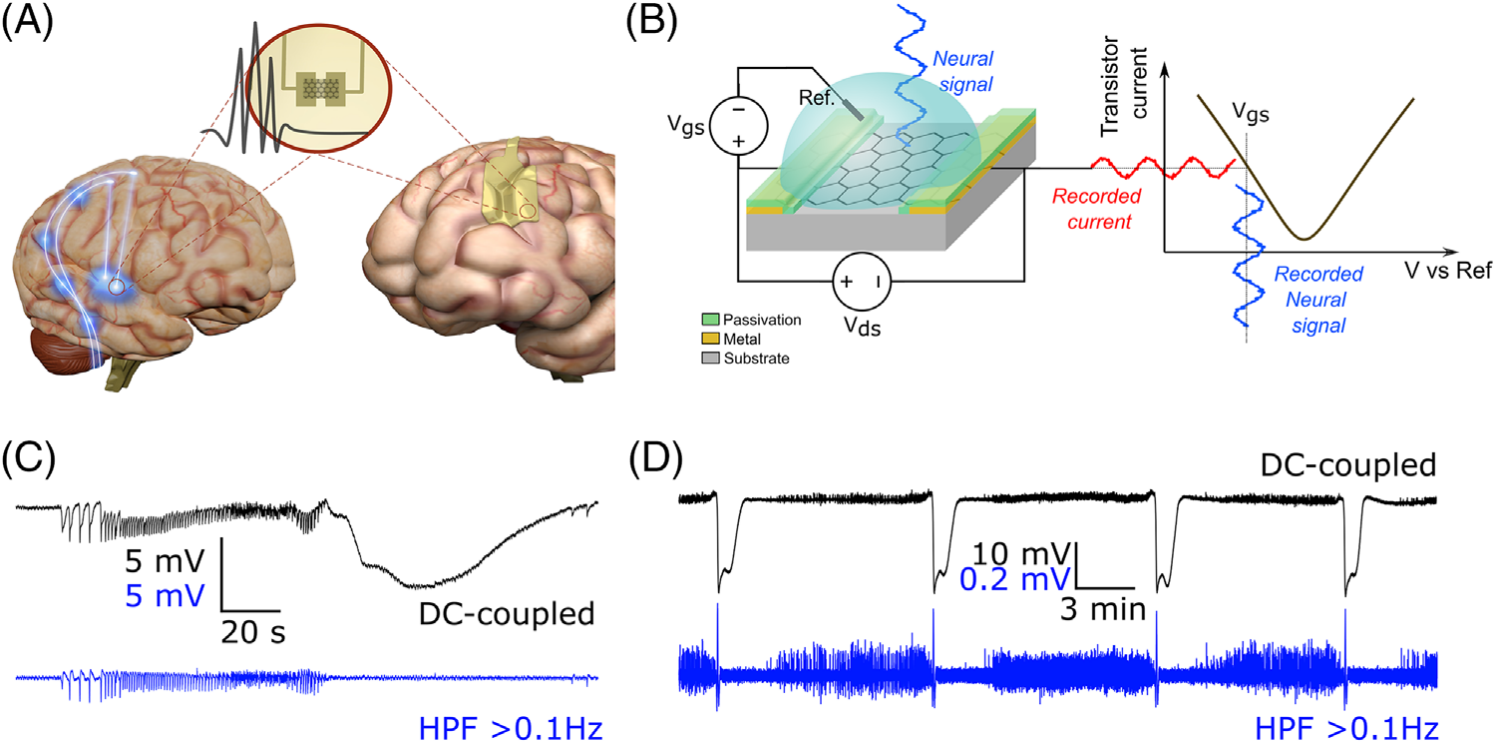
Recording DC‐coupled brain potentials with graphene micro‐transistors. (A) Graphene micro‐transistors can be embedded in surface grids for electrocorticography recordings (right) or in penetrating probes for iEEG (left). (B) Schematic cross‐section of a graphene micro‐transistor showing the driving voltage (Vds) and the operation point selector voltage (Vgs). Current fluctuations in the drain‐source current (Ids) are then converted back to voltage using the transfer characteristics of the transistor (Ids‐Vgs curve). (C), (D) Example DC‐coupled recordings obtained by gSGFETs. DC‐coupled trace (black) and the same trace high‐pass filtered > 0.1 Hz (blue). (C) Recording of a seizure followed by a spreading depolarization using an intracortical array.^11^ (D) Recordings of multiple spreading depolarisations triggered by potassium chloride (KCl) application to the surface of the rat brain.^9^

Solution‐gated field effect graphene micro‐transistors (gSGFETs) take profit from the field effect property of the two dimensional material graphene^7^ to implement a local transduction of neural signals.^8^ gSGFETs consist of a graphene sheet in direct contact with the brain tissue connected by two metal tracks to the recording electronics (Figure 1A and B). The neural signal transduction is produced when brain electrical activity modulates the graphene conductivity and therefore also the current flowing through the transistor when a constant electric field is applied. The high electrochemical stability of graphene permits a stable gain across a wide‐range of frequencies, enabling DC‐coupled recordings with the same fidelity as liquid‐filled glass micropipettes with Ag/AgCl wires^9^ – the current gold standard for DC‐coupled recordings while overcoming their spatial sampling limitations and allowing high‐density mapping.^9^

## 2 PRECLINICAL VALIDATION OF GRAPHENE‐BASED MICRO‐TRANSISTORS DEMONSTRATE THEIR ABILITY TO LOCALISE ULTRASLOW BRAIN SIGNALS WITH HIGH SPATIAL RESOLUTION

The ability to accurately map slow potential changes across large areas of the cortex will have both preclinical and clinical impact. Graphene gSGFETs, flexible, multichannel surface grids (see Figure 1A) have been used to electrographically detect cortical spreading depolarisations (CSD) in vivo (see Figure 1D) either in anesthetised rats^9^ or in awake head‐fixed mice.^10^ Mapping of CSD initiation and propagation permitted quantification of waveform parameters and associated neuronal activity suppression across multiple frequency bands. Spatially localised differences in CSD waveform could be observed despite individual transistors being close to each other (400 μm). This is relevant, as DC‐coupled high‐density mapping using localised electrophysiological biomarkers based on SD waveform (amplitude, duration etc.) can be used to infer the underlying metabolic condition and health of brain tissue in a regionally dependent manner.^4^

In addition to epicortical arrays, highly flexible penetrating probes composed of gSGFETs were developed (see Figure 1A) and shown to record with high fidelity a wide bandwidth of brain signals, from UPS to HFOs.^11^ To further investigate the spatial resolution afforded by multichannel DC‐coupled recordings, current‐source‐density analysis identified sinks and sources of activation through the cortical laminae during chemoconvulsant‐induced seizures in awake mice. High‐pass filtering the data >0.5 Hz to mimic typical AC‐coupled recordings failed to report the same ionic sinks and sources preceding and during the seizure, illustrating the importance of using DC‐coupled recordings for current source density analysis and to avoid misinterpretation of the extracellular potential sinks and sources. A similar layer‐specific localisation of ‘active’ DC shifts preceding seizures could be identified.^11^ Ictal DC shifts can be differentiated into active shifts that precede the seizure and passive shifts that follow the intense neuronal firing at seizure onset.^12^ Active DC shifts are of particular interest for both clinical and basic research, as they provide an electrophysiological biomarker for seizure onset zone localisation,^13^ and they can further provide a quantifiable parameter to investigate the mechanisms underlying seizure initiation.^14^ Chronic implantation of gSGFET probes into the somatosensory cortex of a rat model of absence epilepsy revealed that spontaneous spike‐and‐wave discharges (5–9 Hz) were phase‐coupled to an infraslow oscillation (ISO < 0.1 Hz) most prominent in the superficial layers, suggesting that these ISOs open susceptibility windows for seizure initiation.^11^

SDs can be associated with seizures and vice versa. The dynamic relationship, both temporal and spatial, between these two events is complex and not properly understood. High‐density mapping of both fast seizure activity and ultraslow SDs using gSGFETs promise to shed important insights into these intriguing and clinically important interactions (see Figure 1C for an example recording). Due to their optical transparency, gSGFET arrays are compatible with a range of commonly used imaging modalities and optogenetics^10^ making them particularly attractive tools for preclinical neuroscience research. They also provide a tool that can be used to directly readout in vivo the efficacy of pharmacological^10^ or genetic strategies to suppress or modulate UPS and ISA.

## 3 POTENTIAL CLINICAL APPLICATIONS

Clinical translation of graphene micro‐transistor intracranial probes could offer advantages over current technologies currently used in neurocritical care, epilepsy, intraoperative monitoring and neuroprostheses.

### 3.1 Neurocritical care

The COSBID group have extensively demonstrated that SDs occur clinically following neurotrauma and actively contribute to lesion size in subarachnoid haemorrhage and in post‐traumatic brain injury.^2^ Application of gSGFET arrays to detect SDs in these patients might aid clinical management and provide a direct readout of therapeutic interventions designed to suppress SD initiation and propagation.

### 3.2 Epilepsy

Favourable epilepsy surgery outcome depends upon accurate localisation and resection of the epileptogenic zone. As long‐term seizure freedom after surgery is only around ∼50%,^15^ improvement in technology that more precisely defines surgical margins is warranted. Of note, the few studies that have investigated ictal DC shifts report that these signals co‐localise with seizure onset zones and are more spatially restricted than conventional EEG recordings.^3^ Therefore, routine inclusion of accurate DC‐coupled recordings to surgical monitoring could result in less extensive, yet more effective surgical resections.^16^ Additionally, due to the ability to fabricate high channel density probes with small recording sites, the increased spatial resolution and coverage should allow detection of micro‐seizures, which may be missed using large interspaced electrodes. Beyond presurgical clinical applications, this technology also offers to provide valuable insight into the involvement of UPS post seizure, including investigating the mechanisms underlying postictal generalised EEG suppression (PGES) and sudden unexplained death in epilepsy (SUDEP).^17^

### 3.3 Intraoperative monitoring and neuroprostheses

gSGFET arrays have been shown to preserve their high‐fidelity DC‐coupled recordings when used in multiplexed operation, thus reducing the number of wires and de‐cluttering the surgical site while retaining high spatial mapping with excellent signal‐to‐noise.^18^, ^19^, ^20^ These features will be beneficial for intraoperative monitoring and mapping eloquent cortex. Future applications could include large‐area, high‐density brain mapping devices for neuroprostheses.

In summary, graphene micro‐transistors already contribute to the electrophysiology toolkit in neuroscience research by providing unique capabilities for wide bandwidth recordings, in particular high‐fidelity mapping of ultraslow events. While translation of this technology to the clinic certainly faces many regulatory, adoption and business challenges, we envision a bright potential in clinical applications such as epilepsy, traumatic brain injury, stroke or migraine.

## COMPETING INTEREST

J.A.G. and A.G.‐B. declare financial interest in INBRAIN Neuroelectronics. R.C.W and E.M‐C. have no competing interests.
